# 1st International Symposium on Gait and Balance in MS: Gait and Balance Measures in the Evaluation of People with MS

**DOI:** 10.1155/2012/720206

**Published:** 2012-06-17

**Authors:** Michelle Cameron, Joanne Wagner, Kathleen Zackowski, Rebecca Spain

**Affiliations:** ^1^Department of Neurology, Oregon Health & Science University and Portland VA Medical Center, Portland, OR 97239, USA; ^2^Doisy College of Health Sciences, Saint Louis University, Saint Louis, MO 63104, USA; ^3^Kennedy Krieger Institute, The Johns Hopkins School of Medicine, Baltimore, MD 21205, USA

## Abstract

Gait and balance measures have particular potential as outcome measures in Multiple Sclerosis (MS) because, of the many hallmarks of MS disability, gait and balance dysfunction are present throughout the course of the disease, impact many aspects of a person's life, and progress over time. To highlight the importance and relevance of gait and balance measures in MS, explore novel measurements of gait and balance in MS, and discuss how gait, balance, and fall measures can best be used and developed in clinical and research settings, the 1st International Symposium on Gait and Balance in Multiple Sclerosis was held in Portland, Oregon, USA on October 1, 2011. This meeting brought together nearly 100 neurologists, physiatrists, physical therapists, occupational therapists, nurses, engineers, and others to discuss the current status and recent advances in the measurement of gait and balance in MS. Presentations focused on clinician-administered, self-administered, and instrumented measures of gait, balance, and falls in MS.

## 1. Statement of Need

In the last 30 years, we have made great strides in the treatment of MS. We have developed and brought to market seven disease-modifying medications to reduce the frequency of MS relapses and slow the accumulation of irreversible disability, and many therapies are available to control MS-related symptoms. However, none of these interventions cures MS or halts its progression. One of the significant challenges for developing new and better therapies for MS is that current measures, including the neurological examination, the Expanded Disability Status Scale (EDSS), the Multiple Sclerosis Functional Composite (MSFC), MRI measures, and relapse rates, have limited ability to capture between-relapse and ongoing disease progression in the absence of relapses. As Dennis Bourdette, M.D., Chairman of the Department of Neurology at Oregon Health & Science University (OHSU) said, “what we need [in MS] are measures that are quick, easy, accurate, and sensitive to change—both worsening and improvement—and are understandable [to clinicians]…gait and balance measures have the potential to meet these requirements.” He emphasized the need for communication between neurologists and others caring for people with MS and conducting clinical trials and nonneurologists engaged in developing and testing measures of gait and balance.

## 2. Clinician-Administered Measures

Clinician-administered measures of gait and balance are the measures most commonly used in clinical practice. These measures are generally inexpensive and easy to administer, and many have been validated in people with MS. However, as highlighted by Rebecca Spain, M.D., MSPH, a neurologist from OHSU, because of the diverse goals and perspectives of different clinicians, one measure is not likely to meet every clinician's needs. Neurologists localize neuroanatomically and seek guidance for recommending medications that globally effect MS-related health. In contrast, physical therapists and engineering-based researchers evaluate biomechanical details that underlie gait and balance abnormalities and seek improved methods to direct targeted rehabilitation interventions. 

Francois Bethoux, M.D., a physiatrist from the Cleveland Clinic, noted that many of the currently used clinician-administered measures of gait, including the timed 25-foot walk (T25FW) [1], the 6-minute and 2-minute walk tests (2MWT [2], 6MWT [3]), and the Timed Up and Go (TUG) test [4], can detect changes in gait speed or endurance [5] but do not detect visually obvious improvements in gait quality, or assess important contributors to gait changes such as reduced cognitive function, visual function, upper body trunk control, spasticity, or sensation. Although this limits their utility for guiding selection of specific interventions, these simple clinician-administered measures are still recommended for screening individuals for gait impairment and for standardized assessment of performance over time. 

Susan Bennett, PT, Ed D from the University of Buffalo, described some of the more sophisticated clinician-administered measures of balance, such as the Berg Balance Scale (BBS) [6], the functional reach test, [7] and the recently developed 4-square-step test [8] and mini-BEST test [9]. These measures can differentiate between people with MS who fall from those who do not (BBS [10], 4-square-step test [11]) and can provide information to guide rehabilitation. Dr. Bennett also emphasized the utility of the trunk control test [12, 13] for assessing balance in nonambulatory patients for whom postural control of the trunk is essential for safe and independent transfers.

## 3. Self-Administered Measures 

Marcia Finlayson, OT, Ph.D., from the University of Illinois at Chicago, noted that self-reported measures, also known as Patient Reported Outcome Measures (PROMs), have recently attracted attention from government agencies, including the National Institutes of Health and Food and Drug Administration, because these measures capture directly from patients how they feel or function without interpretation by others. This information from the patient's perspective is important. Self-reported measures of gait and balance can ascertain patients' quality of life, balance confidence, fear of falling, circumstances surrounding fall events, and other important information that cannot be determined by clinical testing or physical instrumentation. These data are usually best captured with diaries, questionnaires, surveys, or web-based methods. 

Joanne Wagner, PT, Ph.D., from Saint Louis University, discussed the MS Walking Scale-12 (MSWS-12) [14], the only PROM specific to walking in MS. This scale assesses self-perceived limitations in walking due to MS. MSWS-12 scores correlate with clinician-administered and instrumented measures of walking, including the EDSS (*r* = 0.65 to 0.84) [14, 15], the T25FW (*r* = 0.46 to 0.65) [14, 15], the 6MWT (*r* = 0.81), [16] the metabolic cost of walking (*r* = 0.64), [17] and free-living accelerometry (*r* = −0.48 to −0.79) [17, 18]. The MSWS-12 also has high test-retest reliability (ICC 0.75 to 0.87) [14, 19], was responsive to change in two medication trials in MS, and has acceptable floor and ceiling effects. A second version of the MSWS-12, adapted from the first version based on Rasch analysis, has been developed and submitted for publication (Hobart, personal communication). Michelle Cameron, M.D., PT, a neurologist from OHSU, discussed the Activities-Specific Balance Confidence (ABC) scale [20] and the Dizziness Handicap Inventory (DHI), [21] the two self-administered measures of balance measures frequently used in people with MS which, although originally developed for other populations, have high test-retest reliability in people with MS (ABC 0.92, DHI 0.90) [22]. They also have fair concurrent validity with each other and identify individuals who fall better than a number of clinician-administered measures including the BBS, the TUG test, the Ambulation Index, and the Dynamic Gait Index [10]. Both the ABC and DHI have also been found to be responsive to the change in trials of rehabilitation interventions. Ylva Nilsagård, PT, Ph.D., from Orebro University in Sweden, focused on using self-reported measures to identify people with MS at increased risk for falling and to determine predictors of falls in people with MS. In a recent study by Dr. Nilsagård, close to 2/3 of MS subjects recorded falls prospectively in a 3-month period [23]. Factors associated with falls were imbalance, weakness, fatigue, and environmental or task-specific factors including being bumped by others, walking in crowds, on ramps, or while carrying objects. 

Self-administered measures of gait and balance in MS provide insight into patients' perspectives about their abilities, are inexpensive to administer, most have minimal floor and ceiling effects, and identify who is at increased risk for falls. However, because these measures do not provide information on neuroanatomical, biomechanical, environmental, or personal factors contributing to gait and balance impairment in MS, or shed light on the impact of activity limitations on an individual's participation or quality of life, they give limited guidance for intervention [24]. The speakers recommended using these measures, in addition to clinician-administered measures, for screening people with MS for gait dysfunction, imbalance, and fall risk, and as outcome measures in trials of interventions expected to have a positive impact on mobility and quality of life. 

## 4. Instrumented Measures

When precise information about gait and balance are needed, instrumented measures are recommended. As related by Fay Horak, PT, Ph.D., and Jessie Huisinga, Ph.D. from OHSU, and Robert Motl, Ph.D., from the University of Illinois at Urbana-Champaign, until recently, sophisticated and bulky equipment installed in specialized motion-analysis laboratories was needed to obtain precise, accurate quantitative gait and balance data. Now, however, wearable sensors housing triaxial accelerometers, gyroscopes, and magnetometers are increasingly used in research settings, and a variety of pedometers and accelerometers are readily available for clinical and home use. These portable devices are also less expensive, require less technical expertise than laboratory-based instruments, and can be used to assess gait and balance in various environments including at home and other “real world” situations. Wearable sensors may have the sensitivity to detect MS-related disability when the clinician cannot [25] and may provide more precise long-term monitoring of disability progression, thus reducing the sample size and trial duration required to determine treatment efficacy.

Despite their great potential, currently, integration of novel instrumented measures of gait and balance into MS clinical practice and research is limited by lack of test protocol and device standardization. We also need to know if these devices are sensitive to changes associated with MS progression and treatment. However, their validation is challenging because it is not clear what measures should be used as a gold standard for comparison. In addition, we do not know how acceptable, feasible, and affordable these new instrumented measures will be for clinical practice and large-scale clinical trials. 

## 5. Relationships between Novel MRI Measures and Instrumented Measures of Gait Impairment

The final speaker of the day, Kathleen Zackowski, Ph.D., OTR, from Kennedy Krieger Institute and Johns Hopkins University, discussed current research on the relationships between conventional as well as novel MRI measures such as magnetization transfer (MT) and diffusion tensor imaging (DTI) with instrumented measures of specific impairments as well as balance and gait changes. She noted that the use of MT and DTI in MS has been growing. These methods allow for a noninvasive evaluation of white matter that is especially useful in diseases where myelin and axonal integrity are disrupted [26–30]. MT indirectly assesses the status of water protons associated with macromolecular structures in tissues such as myelin and is especially useful for studying white matter integrity because white matter has such a high myelin content [31]. However, MT can sensitively quantify white and gray matter abnormalities in both brain and spinal cord [32–34]. DTI-derived indices are sensitive to macroscopic and microscopic tissue disruption and appear to have higher specificity than conventional imaging for areas affected by MS pathology [35]. Using tractography, DTI can define the approximate anatomy of individual white matter tracts within the brain and spinal cord [36]. DTI can identify abnormal values of MRI indices in specific white matter tracts that may underlie clinical disability in MS, and the integration of information derived from other imaging sequences, such as MT, can increase pathologic specificity [34, 37]. DTI abnormalities in the corticospinal tract have been shown to correlate with weakness in MS [38]. These advanced MRI measures may be able to detect and quantify subtle subclinical changes in function impacted by neurorehabilitation and link these with disease pathology. For example, Zackowski et al. [34] demonstrated relationships between instrumented walking velocity measures and DTI and MT indices of the spinal cord in MS. New MRI techniques together with novel instrumented measures of gait and balance may also allow us to define the anatomic substrates of disability and to design specific innovative rehabilitation strategies for people with MS based on the relationships between structure and function. However, larger, more comprehensive studies using a combination of instrumented measures of walking and clinical rating scales, along with more specific MRI indices, such as those from DTI and MT, are needed.

## 6. Discussion 

The symposium concluded with a panel discussion by all of the speakers. The panelists concurred that

1. measures of gait and balance dysfunction should be used throughout the course of MS;
2. currently, available simple clinician and self-administered measures are recommended for screening for imbalance, gait dysfunction, and fall risk;
3. self-administered measures are important for capturing the patients' perspective of their abilities and the effectiveness of interventions;
4. more complex clinician-administered measures and instrumented measures are recommended to guide the selection and development of tailored interventions for walking and balance problems in people with MS and for precise monitoring of change in clinical trials;
5. remaining critical research needs are to identify and standardize the most scientifically valid and practically useful measures to capture the continuum of gait and balance deficits in MS and to understand the relationships between these measures.

Abstracts of posters of primary research presented at this symposium were published in Winter 2011 International Journal of MS Care. The Second International Gait and Balance Symposium in MS is scheduled to be held in Portland, Oregon on October 19th and 20th 2012 and will focus on interventions for gait impairment, imbalance, and falls in people with MS.
